# No Control, No Consumption: Association of Low Perceived Control and Intention to Accept Genetically Modified Food

**DOI:** 10.3390/ijerph19137642

**Published:** 2022-06-22

**Authors:** Shen-Long Yang, Feng Yu, Kai Li, Ting-Ting Rao, Da-Peng Lian

**Affiliations:** 1 School of Humanities and Social Science, Xi’an Jiaotong University, Xi’an 710049, China; yangsl@mail.xjtu.edu.cn (S.-L.Y.); raotingtingpsy@stu.xjtu.edu.cn (T.-T.R.); 2 Department of Psychology, School of Philosophy, Wuhan University, Wuhan 430072, China; 00033940@whu.edu.cn; 3 College of Humanities and Management, Hebei Agricultural University, Huanghua 061100, China; wgldp@hebau.edu.cn

**Keywords:** perceived control, genetically modified food, risk perception, purchase intention, need for structure

## Abstract

Based on compensatory control theory, the aim of this study was to examine the effects of perceived control on people’s acceptance of genetically modified (GM) foods by using both correlational and experimental methods. Compensatory control theory proposes that the lower an individual’s perceived control, the higher their need for structure, order, and certainty. Therefore, based on beliefs about GM foods that make some people less certain that those foods are as safe as traditional foods, we hypothesized that individuals with lower levels of perceived control are more inclined to reject GM foods. The analysis of questionnaire responses in Study 1 revealed that individuals’ sense of control negatively predicted their risk perception of GM foods, while the need for structure played a mediating role. In Study 2, using a between-subject design, we manipulated participants’ perceived control (higher vs. lower) and subsequently measured their risk perception and purchasing preferences for GM foods. The results in Study 2 show that under lower control conditions, individuals recognize higher risks related to GM foods, which, in turn, decreases their willingness to purchase GM foods. These results not only suggest that perceived control is a potential influential personal factor of the acceptance of GM foods but also extend the scope of the application of compensatory control theory.

## 1. Introduction

In recent years, genetically modified (GM) science and technology have burgeoned, similar to the scale of cultivation of genetically modified crops. According to the International Service for the Acquisition of Agri-biotech Applications (ISAAA), the area planted with GM crops worldwide has increased from 1.7 million hectares in 1996 to 189.8 million hectares (112-fold) in 2017 [1]. GM agricultural products often have advantages over traditional agricultural crops, including better taste and aesthetics and prolonged freshness. However, people’s acceptance of GM food products is relatively lower [2]. The European Union governments, in particular, exercise extremely strict control over GM crops, and consumer acceptance of these commodities is very low [3,4,5]. In China, a nationwide sample survey showed that only 12% of consumers were willing to buy genetically modified foods [6]. In general, people still choose GM foods less often than traditional agricultural products, sometimes harboring extreme emotions toward them.

The reasons behind the low GM food acceptance remain not very clear. Although some key factors, including perceived risks and benefits, trust in authority, knowledge, and subjective norms, were found to influence people’s attitudes toward GM food [4], more research is needed to explore the predicting variables associated with our attitudes about GM food. The existing research pays more attention to examining the complete information processing model of consumers when they understand GM food and decide to purchase it, and then explains the cognitive style of people’s attitude toward GM [7,8]. For example, some researchers hold that people tend to oppose GM products because they lack sufficient scientific knowledge but believe they have a wealth of knowledge about GM food [9]. Similar findings were also found in China, showing that consumers do not trust the claims made by the government and scientists, which constitutes a reason people do not choose genetically modified foods [6]. In addition, some studies have proposed integrated models of information processing to predict people’s propensity to buy genetically modified foods [10,11]. Risk perception, in particular, has been studied relatively often [12,13] and is considered a more important predictor of people’s opposition to GM food than perceived benefits [11,14,15]. However, it should be noted that for the same GM food, people’s perceived risk may also depend on individual differences, such as certain personality traits or state variables, potentially making people more inclined to either ignore or magnify the risks. Some studies also look at individual difference factors, such as food neophobia [16], disgust sensitivity, and cultural values [12]. However, existing studies have not fully explored the factors of these individual differences, and the existing conclusions include people’s acceptance of other new technologies (rather than only based on genetically modified foods). Given the perspective that individual difference factors may underlie risk perception, we aim to investigate perceived control (an individual difference factor that few previous studies have focused on regarding its effect on risk perception and the acceptance of GM foods) and its potential effect on people’s GM food selection from the perspective of compensatory control theory.

As an individual difference factor, people’s perceived control is a variable with both stability and variability [17,18]. From the existing research, although some studies see it as a stable variable of individuals, it can also be treated as a state variable and can be manipulated by experimental priming [19]. As a basic individual difference concept, perceived control reflects the relationship between an individual and the external world, and can predict many positive mental and psychological outcomes [20]. However, previous studies have not examined the relationship between perceived control and the acceptance of GM foods.

Therefore, this study aims to propose and explore a variable that affects individuals’ attitudes toward GM foods, that is, perceived control, which previous studies have not examined. While previous studies have identified a key predictor, i.e., risk perception, for people’s acceptance of GM foods, this study suggests that risk perception can be influenced by individual differences, such as perceived control. This will help enrich the accumulation of research on the consumption and cognition of GM foods and also expand our understanding of the effects of perceived control. In practice, further clarifying the influencing factors and individual differences in people’s perception of GM risks will also help the government and scientists better conduct scientific and educational propaganda for specific groups, guide target groups to accept GM foods more efficiently, and promote the public endorsement and consumption of GM crops.

## 2. Theoretical Framework and Hypotheses

Although knowledge of the benefits of GM food can positively predict consumers’ attitudes toward it, studies have found that, for many consumers, the related risks often outweigh the benefits; this finding suggests that consumers’ decisions are predicated more on the perceived risk than the perceived benefits [11,14]. Essentially, risk (rather than benefit) information exerts a greater and more prolonged impact on consumer attitudes [15]. As a result, those who do not choose genetically modified food may be aware of its advantages but struggle to accept the potential disadvantages. Furthermore, the media and online folk opinions have created hype around the potential risks of GM foods, leading many to believe that GM food is not 100% safe and presents an uncertain risk [21].

Although the evidence is unclear, we propose that there are likely vital individual differences that breed intolerance for the uncertain risks inherent to GM foods, leading people to reject them. For example, some people are more sensitive to risk and less able to handle potential risks than others with low risk perception and high risk tolerance [22,23]. In this study, we mainly focus on the influence of perceived control on the acceptance of GM products by individuals.

Perceived control, or sense of control, refers to an individual’s perception of their ability to control events and the extent to which they feel subjected to external constraints [24]. It is understood as a relatively stable individual difference variable but can also be manipulated under certain conditions to some extent [17,18]. A sense of control is of great significance to human beings; gaining control of the external environment counts among the most basic human motivations [25]. Higher perceived control can predict a range of positive physical and mental health outcomes [26,27]. However, in daily life, people face many uncertainties, and their need to control the outside world is not always satisfied. Moreover, some people have an inherent relatively low sense of control. According to compensatory control theory, lower perceived control evokes one’s need for structure, which means they demand certainty, order, and predictability [28].

Compensatory control theory explains the sense of control as a basic human need that helps individuals see the environment as stable and safe [29]. However, people often face uncontrollable situations, resulting in one’s need to control the outside world not always being realized. Therefore, compensatory control theory argues that an individual’s need for structure is likely to increase to compensate the lack of personal control [30]. Previous studies have supported this argument through empirical evidence in many aspects. For example, those with relatively lower perceived control tend to prefer rhythmic music to atonal music [31]. Researchers also found that individuals with lower levels of perceived control exhibit more interest in ordered and structured theoretical ideas [32], even conspiracy theories [33] or fake news [34]. In the context of the COVID-19 pandemic, people with a lower sense of control also had a higher need for structure [35]. In short, compensatory control theory holds that individuals with a lower sense of control will prefer structured, orderly, and predictable social and physical patterns to compensate for their temporary low sense of control.

Moreover, the tendency to need structure (due to an insufficient sense of control) is reflected in consumer behavior. Individuals with a lower sense of control exhibit a higher preference for products that are structured and orderly. One mobile-phone-purchase experiment [36] found that participants in the experimental lower-control group paid more attention to whether the mobile phone on offer could bring order to life. Similarly, previous research also found when consumers’ perception of control is threatened, they prefer utilitarian goods [37] and products with more controllability [38] to fulfill their need for structure.

**Figure 1:**
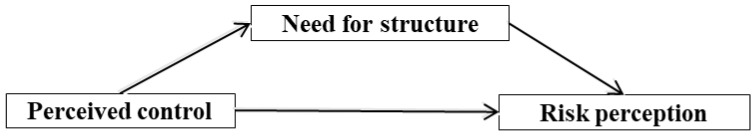
Hypothesized relationship among perceived control, need for structure, and risk perception.

Based on compensatory control theory and research findings, we can speculate that people’s sense of control predicts how comfortable they are being in uncertain circumstances or having a lack of clear structure in various tasks or situations. The less they have a sense of control, the more they prefer certainty, order, and predictability. Compared to “traditional” food, most people’s understanding or thinking about GM food is more ambiguous. Thus, for people with a low sense of control, GM foods cannot meet their need for structure. Consequently, it is perceived as riskier. Therefore, the present study posits (see also Figure 1):

**H1:**  *An individual’s perceived control can negatively predict their risk perception of GM foods*.

**H2:**  *The need for structure plays a mediating role between perceived control and risk perception of GM foods*.

**Figure 2:**
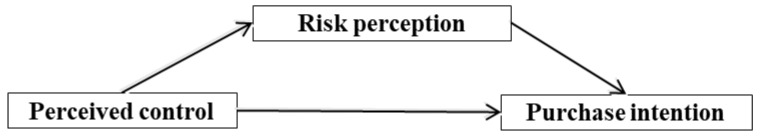
Hypothesized relationship among perceived control, risk perception, and purchase intention.

In addition, the perceived risk of GM foods positively predicted individuals’ tendency to buy fewer GM foods [39]. Hence, we want to explore whether people with a lower sense of control would buy fewer GM foods. Since the purchase of GM foods is also inconsistent with the demand for structure, we assume that (see also Figure 2):

**H3:**  *Individuals’ perceived control can positively predict their willingness to buy genetically modified food*.

**H4:**  *Risk perception mediates the relationship between perceived control and GM purchase intention*.

We tested the above hypotheses through two studies: In Study 1, we tested H1 and H2 (dependent variable: risk perception of GM food), and in Study 2, H3 and H4 (dependent variable: intent to purchase GM food) were examined. We aimed to investigate risk perception and purchasing behavior to understand whether the individuals’ acceptance of genetically modified food can be explained from a psychological and behavioral perspective. Our study used Chinese participants since genetically modified crops are commercially grown in China, and the general public is familiar with them [40], being exposed to them almost daily.

## 3. Study 1

The purpose of Study 1 is to test H1 and H2, that is, the predictive effect of perceived control on the risk of GM food perceived by individuals and the mediating effect of the need for structure. To this end, we collected data through a questionnaire method and observed the relationship between variables.

### 3.1. Study 1 Methods

#### 3.1.1. Participants

We conducted a paper-and-pencil survey on college students in a Chinese university in Shaanxi province, and 436 undergraduate students were recruited to fill in the questionnaire voluntarily. All participants were fully informed that their anonymity was assured, why the research was being conducted, and how their data would be used. Furthermore, we told every participant that no potential risks or possible discomfort had been found so far, and that if they were uncomfortable with the study, they could choose to finish the participation at any time. Once they had been fully informed of the details, continued participation was taken as informed consent. To thank them for their time, each participant who provided valid responses received CNY 3. For attention checks, we included two questions to identify whether each participant’s data were valid, namely, “Please choose ‘strongly agree’ for this question” and “Please choose ‘strongly disagree’ for this question”, which confirmed whether the participants had read the questions carefully. Data from participants who failed to answer these questions correctly were deleted. Participants who did not complete the questionnaire were also excluded. In total, 35 participants with invalid data were excluded, leaving a final sample of 401 (245 male, *M*_age_ = 19.52, *SD* = 1.10), which was higher than the recommended sample size (*N* ≈ 250) for obtaining stable coefficients based on the average effect size (*r* ≈ 0.20) in social and personality psychology [41,42].

#### 3.1.2. Measures

##### Perceived Control

Perceived control was measured using the Perceived Control Scale [24]. A 7-point Likert scale (1 = strongly disagree, 7 = strongly agree) was adopted, and the average score of 12 items (for example, “I can do just about anything I really set my mind to”) reflected the level of perceived control. The higher the score, the higher the participant’s sense of personal control. In this study, the Cronbach’s *α* was 0.83.

##### Need for Structure

Personal need for structure was measured with an 11-item scale (example item: “I become uncomfortable when the rules in a situation are not clear”) developed by Neuberg and Newsom [43]. Participants responded on a 6-point Likert scale (1 = strongly disagree, 6 = strongly agree). The average score of the 11 items reflected the “need for structure” score. Higher scores represented a higher need for order, structure, and certainty. The Cronbach’s *α* was 0.75 in this study.

##### Perceived GM Food Risk

We applied Chen and Li’s [44] 3-item (example: “Eating transgenic technology food can cause harm to the health of my family”) scale to measure this aspect. Participants responded on a 5-point Likert scale (1 = strongly disagree, 5 = strongly agree). We took the average score of three items as the total score; higher scores represented higher perceived risks. In this study, Cronbach’s *α* was 0.74.

### 3.2. Study 1 Results

#### 3.2.1. Descriptive Statistics and Correlations Analyses

Means, standard deviations, and Pearson correlations were calculated (Table 1). Perceived control was negatively correlated with the need for structure (*r* = −0.23, *p* < 0.001) and risk perception (*r* = −0.19, *p* < 0.001), supporting H1. The need for structure was positively correlated with risk perception (*r* = 0.14, *p* = 0.004). The results fitted the basic condition for mediation analysis among the three variables.

#### 3.2.2. The Mediating Role of Need for Structure

**Figure 3:**
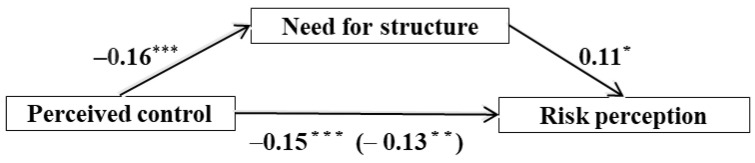
Mediation model. *N* = 401; * *p* < 0.05; ** *p* < 0.01; *** *p* < 0.001.

We used the PROCESS macro for SPSS 23.0 (Chicago, IL, USA) (Model 4) developed by Hayes [45] to test the mediation model among perceived control, need for structure, and risk perception. As shown in Table 2 and Figure 3, the results exhibited that perceived control was significantly associated with risk perception, *b* = −0.15, *t* = −3.83, 95% confidence interval (CI) = [−0.22, −0.07], *p* < 0.001, and perceived control was negatively associated with the need for structure, *b* = −0.16, *t* = −4.71, 95% CI = [−0.23, −0.10], *p* < 0.001. When perceived control and the need for structure were entered simultaneously into the regression, the association between perceived control and risk perception was lower, although still statistically significant (*b* = −0.13, *t* = −3.27, 95% CI = [−0.20, −0.05], *p* = 0.001), and the association between the need for structure and risk perception was also statistically significant (*b* = 0.11, *t* = 2.08, 95% CI = [0.01, 0.22], *p* = 0.038), thus supporting H1 and H2. The ratio of the indirect effect to the total effect was 12.75%.

### 3.3. Study 1 Discussion

The results showed that Study 1 supported H1 and H2. The level of an individual’s sense of control was negatively associated with the perceived risk of genetically modified food. The lower the sense of control, the more risk they felt the GM food had. Moreover, the research data supported the mediating role of the need for structure: individuals with a low sense of control would have a stronger demand for structure, order, and certainty. Based on this mechanism, they felt that GM food was riskier than non-GM food. These results are aligned with the perspectives of compensatory control theory but are based on the logic of the correlation method. Thus, we cannot infer a causal relationship between the sense of control and people’s perception of the risks of GM food. Moreover, can the influence of the perceived control on risk perception be further extended to people’s willingness to buy GM food? As these were questions that Study 1 could not answer, we proceeded to supplement the data by conducting Study 2.

## 4. Study 2

The purpose of Study 2 was to test H3 and H4 and investigate whether the effect of the perceived control on risk perception could be further extended to the intent to purchase GM food. Specifically, Study 2 examined the predictive effect of perceived control on the purchasing intent and whether perceived risk played a mediating role. Since Study 1 was not adequate for revealing the causal relationship between perceived control and risk perception, we examined the causal relationship using an experimental design in Study 2 to further clarify their relationship.

### 4.1. Study 2 Methods

#### 4.1.1. Participants

We recruited undergraduate students from a university in Shaanxi province in China to participate in the experiment. G*power analysis showed that when the effect size *(d)* was 0.5, the total sample size of about 102 participants was needed to achieve 80% power [46]. Hence, we recruited a few more participants than the recommended amount (*N* = 130). All participants were fully informed that their anonymity was assured, why the research was being conducted, and how their data would be used. We told every participant that no potential risks or possible discomfort had been found so far, and that if they were uncomfortable with the study, they could choose to finish the participation at any time. Once they had been fully informed of the details, continued participation was taken as informed consent. To thank them for their time, each participant who completed the experiment received CNY 5. For attention checks, we included two questions to identify whether each participant’s data were valid, namely, “Please choose ‘strongly agree’ for this question” and “Please choose ‘strongly disagree’ for this question,” which confirmed whether the participants had read the questions carefully. Data from participants who failed to answer these questions correctly were deleted. Participants who did not complete the whole procedure were also excluded. In total, 15 participants with invalid data were excluded, leaving a final sample of 115 (62 male, *M*_age_ = 20.17, *SD* = 1.68).

#### 4.1.2. Experimental Procedures and Materials

We adopted a single factorial between-groups design with independent (experimentally-induced perception of control—high vs. low), dependent (intention to consume GM food), and mediating (risk perception) variables.

Participants were randomly assigned to high or low perceived control priming conditions at the laboratory so that they could temporarily experience different states of perceived control. Thereafter their perceived control state was assessed. Then their perceived risk and intent to purchase GM foods were tested and the researcher told them that the questionnaires were for another study that was not connected to the former task. Each participant who had completed the entire test received a participation fee.

We manipulated perceived control with a recall task [47], requiring participants to recall and document an event where they had a sense of full control (higher perceived control condition) or a sense of no control (lower perceived control condition) to temporarily experience different perceived control states. Then we examined the manipulation result with the Perceived Control Scale (the same as Study 1). Cronbach’s α was 0.84 in this study.

Thereafter, the participants’ perception of GM risk and their willingness to purchase GM food was measured. The measurement of GM risk perception was consistent with Study 1. A questionnaire compiled by Chen and Li [44] was used (α = 0.79 in Study 2). Next, we measured individuals’ intent to purchase GM food using a three-item scale [48] (example: “I will consume GM food in the future”), requiring them to respond on a 5-point Likert scale. We took the average score of three items as the total score; higher scores represent higher levels of intent. Cronbach’s *α* was 0.84 in this study. Finally, we obtained the participants’ demographic information.

### 4.2. Study 2 Results

#### 4.2.1. Manipulation Check

Firstly, we conducted a manipulation check of the effect of the perceived control priming. An independent samples *t*-test was conducted to examine the difference in the average level of perceived control between the two groups. The results confirmed that the participants induced to experience higher level control reported a higher sense of control (*M* = 4.69, *SD* = 0.82) than those induced to experience a lower level control status (*M* = 4.26, *SD* = 0.83), *t* (113) = 2.80, *p* = 0.006, Cohen’s *d* = 0.52, indicating that the experimental manipulation was effective.

#### 4.2.2. Descriptive Statistics and Correlations Analyses

Next, means, standard deviations, and Pearson correlations were calculated (Table 3). Perceived control was significantly correlated with risk perception (*r* = −0.26, *p* = 0.006) and intent to purchase GM food (*r* = 0.24, *p* = 0.011). Purchasing intent was significantly correlated with risk perception (*r* = −0.60, *p* < 0.001). The results fitted the basic condition for mediation analysis among the three variables.

#### 4.2.3. The Mediating Role of Risk Perception

**Figure 4:**
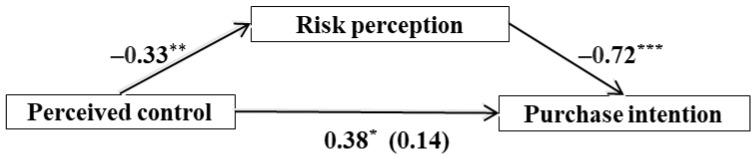
Mediation model. *N* = 115; * *p* < 0.05; ** *p* < 0.01; *** *p* < 0.001.

We used the PROCESS [45] macro for SPSS 23.0 (Model 4) to test the mediating model among perceived control, risk perception, and the purchase intention of GM foods. As shown in Table 4 and Figure 4, the results exhibited that the total effect of perceived control on purchasing intent was significant (*b* = 0.38, *t* = 2.58, 95% CI = [0.09, 0.67], *p* = 0.011) and perceived control negatively predicted risk perception (*b* = −0.33, *t* = −2.82, 95% CI = [−0.56, −0.10], *p* = 0.006). When perceived control and risk perception were entered simultaneously into the regression to predict purchase intention, the association between perceived control and purchase intention was diminished (*b* = 0.14, *t* = 1.13, 95% CI = [−0.11, 0.38], *p* = 0.263), and the association between risk perception and purchase intention was statistically significant (*b* = −0.72, *t* = −7.48, 95% CI = [−0.91, −0.53], *p* < 0.001). The ratio of the indirect effect to the total effect was 62.98%.

In summary, Study 2 supported H3 and H4. The level of individuals’ perceived control positively predicted their willingness to purchase GM foods. That is to say, the lower their sense of control, the less likely they were to buy GM foods. Moreover, the research data supported the mediating effect of risk perception. Individuals with lower perceived control would be more concerned about the risk of GM foods. Based on this mechanism, they would be more reluctant to buy GM foods. This also fitted our hypothetical prediction.

## 5. Discussion

In parallel with rising GM food production and sales, studies have increasingly focused on people’s attitudes and purchase intentions regarding GM foods. Highlighting the influencing factors and psychological mechanisms behind this, researchers have focused on risk perception’s influence, counting it as at least one of the most important predictors of anti-GM food views [11,49]. Some studies found that risk (rather than benefit) perception is more effective in predicting anti-genetic attitudes. Moreover, through risk perception’s mediating role, other factors may indirectly influence GM acceptability. Still, questions arise about the individual difference variances in GM food risk perception [50,51]. Investigating whether individual difference factors affect one’s attitude toward GM food is important because these individual difference variables enable sellers to adopt different marketing strategies to communicate with different groups of people.

Our research examined the effect of perceived control on individuals’ attitudes about GM food. We conducted two studies. Both studies consistently found that people with a lower perceived control were more negative about GM foods than those with higher perceived control. As perceived risk has repeatedly been documented as a predictor of attitudes toward GM foods generally [52], our research focused on the relationship between individuals’ risk perceptions and purchase intentions concerning GM foods.

Study 1 found that perceived control positively predicted people’s risk perception of GM foods. The lower the perceived control, the higher people’s perception that GM foods involve risk. Here, the mediating mechanism was the individual’s need for structure. Thus, given an insufficient sense of control, the individual has a pressing need to perceive things around him as orderly and predictable. Driven by this motive, individuals feel that genetically modified food involves more risks. Study 2’s experimental method revealed the causal relationship between perceived control and the perception of GM risk and extended this effect to the intent to purchase GM foods. We found a mediating relationship between perceived control, risk perception, and the purchase intention of genetically modified food: perceived control could influence the purchasing intent by impacting the risk perception of GM foods. Specifically, individuals in the low sense of control condition considered GM foods to be higher risk foods and were less willing to buy them than the participants in the high sense of control condition. Thus, our study revealed that perceived control—an individual difference factor—could influence the risk assessment and purchasing intent toward genetically modified food. Additionally, this study also found that the risk perception of GM food could predict people’s purchase intention, indicating that risk perception was an important predictor of consumption decisions, consistent with the conclusions of previous studies [11,12,13,14,15].

Moreover, the results of this study indicate that, due to relative uncertainty regarding GM food, people with a lower sense of control are more likely to perceive it as high risk (compared with traditional food); thus, they are less willing to accept GM food products. The psychological mechanism of this effect entails individuals with a lower sense of control having a greater need for an ordered, structured, and certain external world to compensate for that limited sense of control [28,29]. Consequently, they become more anxious and have fewer positive attitudes toward things that do not offer certainty. Thus, our results confirm the influence of risk perception on the purchase of GM food and reveal why different people have different perceptions of the risk of genetically modified food, explaining it from the perspective of the perception of personal control.

Numerous previous studies have looked at the trade-off between risk and benefit [11,12,13,14,15] or the impact of knowledge [9,10] on individuals’ acceptance of GM foods. These studies help us better understand people’s psychological processes when buying GM foods, and perceived risk is considered to be one of the most critical factors to predict people’s attitude toward GM products. However, there may be more fundamental factors such as individual differences and psychological structures at work behind these psychological processes. Although recent studies considered this and examined several individual difference variables [12,16], more factors remain to be revealed. Along this line of thought, based on the theory of compensatory control, this study found that the sense of control may also be a basic individual difference factor that can influence this process. This is a conclusion that few past studies have addressed. An individual’s attitude toward GM food is largely derived from their perceived risk, while their perceived risk is not entirely based on the possibility of risk objectively, and partly depends on their personal factors. Of course, there are far more potential research perspectives on individual differences in attitudes toward GM foods, which can be explored in the future.

In summary, regarding the research on attitudes toward GM food, our study verifies the important role of risk perception that previous studies believed. Moreover, we extend these streams of research by proposing and examining that a personal sense of control can be understood as a more fundamental factor influencing risk perception. At the same time, based on the finding that individuals with a lower sense of control have a relatively higher need for structure, and individuals with a higher need for structure tend to be more sensitive to risk, our study explains why a personal sense of control is related to the risk perception of GM food. These are innovative findings for the research field.

Furthermore, this study contributes to the domain of compensatory control. Compensatory control theory was proposed in 2008 [19,47], and copious amounts of research have supported the theory. The theory explains that when the level of control is lower, individuals have a higher need for a structured and ordered external environment. This kind of compensatory control can manifest in many ways, including supporting candidates who can provide order and stability to society at the political level [36] and, at a consumer level, preferring products that can bring order to life [37], and so on. This study extended the application scope of the compensatory control theory showing that it could explain the attitudes and behaviors of individuals toward genetically modified food. Due to their uncertainty about GM food, which does not meet the need for order and structure, people with a lower sense of control will perceive it as presenting a greater risk than other food, and they are more inclined to reject GM food. This result not only fits within compensatory control theory but also provides new evidence supporting it.

This research also has some practical value. Past surveys have found that people generally dislike GM foods and are less willing to buy them [3,4,5,6]. In addition to understanding this situation in terms of knowledge and cognitive tendencies, it may be necessary to consider individual differences in the population. From the government’s point of view, their scientific dissemination of GM foods should be more targeted at individuals with a low sense of control, and their efforts should be made to improve the perceived control of its citizens, especially those who do not accept GM technology. For the marketing strategy of GM food, the sellers can take individuals with a higher sense of control as their main target customers. For example, they can devote more sales effort to individuals with a higher socioeconomic status, who generally have a higher sense of control, according to previous studies [53].

There were some deficiencies in the present study. First, the research samples were college students. Previous studies have found that the effect of perceived control is consistent in both undergraduate and non-undergraduate adult samples [28,47,54]. Therefore, this study carried college students as samples to reveal the relationship between individuals’ sense of control and attitudes toward GM food. However, there may be latent issues of sample representativeness. Hence, future studies should test the hypotheses with a broader range of participants from various backgrounds such as people of different ages, levels of education, and occupations; though we expect these studies with different samples to replicate the results of this study. Second, this study was only based on the compensatory control theory. There may be other mechanisms through which a sense of control influences attitudes toward GM foods. For instance, past studies have found that individuals with a low sense of control are generally less healthy [20], and lower fitness may also be a reason for people to reject GM crops, which can be considered in a future investigation. In addition, more personality and individual difference factors can be tested to explore their relationship with people’s attitudes toward GM food. For example, an individual’s tendency to believe in conspiracy theories, which past research has found to be a variable associated with a lower sense of control [28,33], may also serve as a psychological basis for people not accepting GM products. This perspective needs to be further explored in future research. Third, our study did not further investigate the boundary conditions under which the perception of control affects the acceptance of transgenic organisms. For example, there are many kinds of GM foods, some transferring DNA from an organism in the same species and others transferring DNA from a different species. Perhaps the former is more likely to satisfy people’s need for structure, therefore appearing more acceptable to people with a lower sense of control. Fourth, future studies could consider how to change consumers’ attitudes toward GM foods by improving their sense of control. Some studies found that individuals’ perceived control can be changed [54]. Thus, researchers can conduct a study to test the ability to enhance people’s acceptance of GM foods by changing their perception of control over outside situations. Finally, future research could integrate the findings of this study more deeply into sales practices. Since this study only focuses on purchase intentions, the researchers may further examine the consumers’ sense of control and other individual difference characteristics through questionnaires, experiments, and big data methods in the actual sales of GM products to make the research conclusions more ecologically valid.

## 6. Conclusions

The results of this study strongly suggest that people with a lower sense of control are more likely to perceive GM foods as risky, and their personal need for structure plays a mediating role in that perception. Additionally, the reluctance to buy GM foods is greater among individuals with lower perceived control who identify GM foods as “risky”.

## Figures and Tables

**Table 1:** Study 1 descriptive statistics and correlations (*N* = 401).

Variables	M	SD	1	2	3	4
1. Gender	0.61	0.49				
2. Age	19.52	1.10	0.04			
3. Perceived control	4.51	0.78	−0.09	0.04		
4. Need for structure	3.98	0.56	−0.07	0.08	−0.23 ***	
5. Risk perception	2.88	0.60	−0.07	−0.05	−0.19 ***	0.14 **

** *p* < 0.01; *** *p* < 0.001. Gender: male = 1; female = 0.

**Table 2:** Study 1 mediation effects analyses (*N* = 401).

Outcome Variable	Independent Variable	*R* ^2^	*b*	*t*	95% CI
Risk perception	perceived control	0.04	−0.15	−3.83 ***	[−0.22, −0.07]
Need for structure	perceived control	0.05	−0.16	−4.71 ***	[−0.23, −0.10]
Risk perception	need for structure	0.05	0.11	2.31 *	[0.01, 0.22]
	perceived control		−0.13	−3.27 **	[−0.20, −0.05]

* *p* < 0.05; ** *p* < 0.01; *** *p* < 0.001. Perceived control: higher = 1; lower = 0.

**Table 3:** Study 2 descriptive statistics and correlations (*N* = 115).

Variables	*M*	*SD*	1	2	3	4
1. Gender	0.54	0.50				
2. Age	20.17	1.68	0.05			
3. Perceived control	0.50	0.50	0.04	−0.08		
4. Risk perception	3.00	0.65	−0.16	0.17	−0.26 **	
5. Purchase intention	2.92	0.80	0.17	−0.06	0.24 *	−0.60 ***

* *p* < 0.05; ** *p* < 0.01; *** *p* < 0.001. Gender: male = 1; female = 0. Perceived control: higher = 1; lower = 0.

**Table 4:** Study 2 mediation effects analyses (*N* = 115).

Outcome Variable	Independent Variable	*R* ^2^	*b*	*t*	95% CI
Purchase intention	perceived control	0.06	0.38	2.58 *	[0.09, 0.67]
Risk perception	perceived control	0.07	−0.33	−2.82 **	[−0.56, −0.10]
Purchase intention	risk perception	0.37	−0.72	−7.48 ***	[−0.91, −0.53]
	perceived control		0.14	1.13	[−0.11, 0.38]

* *p* < 0.05; ** *p* < 0.01; *** *p* < 0.001. Perceived control: higher = 1; lower = 0.

## Data Availability

Data will be provided by the authors on request.
